# A Reply to Messieurs Esquirol's and Falret's Objections to Dr. Burrows' Comparative Proportions of Suicides in Paris and London

**Published:** 1822-12

**Authors:** G. M. Burrows


Art. VI.—*A Reply to* Messieurs Esquirol's *and* Falret's *Objections to* Dr. Burrows' *comparative Proportions of Suicides in Paris and London.* By G. M. Burrows, M.D. F.L.S. &c.

Dr. Esquirol, in the 53d volume of the "Dictionnaire des Sciences Médicales," article *Suicide*, has entered into an examination of the grounds on which, in my "Inquiry into certain Errors relative to Insanity," I have decided that self-destruction is more frequent in Paris than in London.

As the contrary has been a favourite axiom with foreign, especially French, writers, it was to be expected that any attempt to controvert it would not long remain unnoticed. Accordingly, not only Dr. Esquirol, but his élève Dr. Falret, in a work entitled "De l'Hypochondrie et Suicide, 1822," following his master's example, has attacked both the premises and deductions I have adopted.

A regard for truth, as well as respect for my own integrity and understanding, which are equally impugned by the observations of these authors, impose on me the obligation of a reply.

The knowledge that suicide was more prevalent in the French than in the British metropolis, I casually gleaned, while writing some observations on the comparative mortality of the two cities. This paper was published in the "London Medical Repository, for December 1815," accompanied by some remarks on a circumstance so opposite to received opinions. Therefore, when preparing my "Inquiry," the object of which is principally to correct errors relative to insanity, physical, moral, and civil, the subject I had before only incidentally touched became one of legitimate investigation.

Indeed, I considered the fact fit matter for history, and was, therefore, more careful to establish it by unquestionable authority. I had no design to attach reproach or odium on any nation. As a Christian and a British citizen, I felt it to be a duty to disprove the accusation, that Englishmen were more addicted to the crime of suicide than the inhabitants of other countries. But I never meant even to hint that the frequency of it was diminished among us: nay, I have concluded with saying, that the comparison merely proves us "to be lowest in the scale of impiety; for suicide, it must be confessed, is a vice still much too common in England."—*Inq.* p. 93.

For Dr. Esquirol I entertained much personal respect, and have given him proofs of it. I have visited La Salpêtrière, where he so ably seconds Dr. Pinel, and his own private establishment for lunatics, and know that his views are most humane. I think, too, medical science highly indebted to him for his pathological researches and various writings on the subject of insanity. With deep regret, therefore, it is that I perceive, in his comments on my statements relative to suicide, a spirit unworthy his high character. I would rather ascribe the many perversions, omissions, and misrepresentations of my text, to ignorance of the English language, than to a wilful design to suppress truth. Indeed, the blunder in the title of my book (Inquiry relative *of to* Insanity,) favours that conclusion. But, unluckily, Dr. Esquirol, in referring to other English authors, has evinced a very correct knowledge of their works. Consequently, can I interpret his reading of mine in any other light than intentional?

1. Dr. E. says, that I ought rather to accuse English than French historians and physicians with charging insanity as being endemical in England, especially Smollet.

Smollet was an hypochondriac; and Cheyne, to whom I chiefly attribute this character of us, had his private views in obtaining belief that the "spleen" was a popular malady among English people. Even if this charge were true in relation to the period when these authors wrote, near a century ago, how does that apply to the present age? Has no revolution, in the course of that time, occurred in the religious, moral, or political state of France, which should have induced insanity and suicide more frequently than in England? But, I repeat, I do not deny the frequency of insanity or suicide in England.

2. Again; Dr. E. asserts, (*Dict. des Sc. Med.* p. 276,) that the British Parliament, in 1815, loudly proclaimed that the insane were more numerous in England than on the continent.—The British Parliament never made any such declaration, either then or at any other period.

3. To understand clearly Dr. Esquirol's further objections, it is necessary to premise that, in my comparative table of the proportion of suicides to the population of different European cities in 1817, Paris and London stand thus:

| | Suicides. | Population. | Proportion. |
|---|---|---|---|
| Paris------ | 300 ---- | 700,000, | or 0.42 in 1000, |
| London---- | 200 ---- | 1,000,000, | or 0. 2 in 1000. |

4. To invalidate these proportions, Dr. Esquirol has recourse to various arts. He pretends to quote an entire passage, containing my proofs and reasoning, in support of them. But it is impossible to conceive a passage to be more garbled and misrepresented. To judge fairly, let the translation in the Dictionnaire des Sciences Medicales, tome 53, p. 277, be compared with the original in the Inquiry, p. 87—89. I will give one specimen: I have written "The *population*, within the London Bills of Mortality, much exceeds that of the department of the Seine, in which is included Paris: the difference may be about as 10 to 7." Dr. E. translates this—"La *mortalité* (not *population*,) à Londres est beaucoup plus forte que celle de tout le département de la Seine, qui renferme Paris: elle est comme 10 à 7."

5. From the relèves of the department of the Seine, I learnt that, in 1817, the number of suicides exceeded 300. The number entered in the London Bills of Mortality, for the same year, is 40. Of 100 entered as drowned, I have assumed that 40 may have met a voluntary death. Of the 120 registered as having died insane, there are doubtless some who have committed suicide: but, that I might cover all possible omissions, I have reckoned the whole 120 as suicides; and thus collectively made up the 200 inserted in the above comparative table.

This, I admit, is a loose way of reckoning, for which I may be justly blamed by English moralists; but I did not imagine that Dr. Esquirol would object that I support the proportion of 200 suicides in London by a series of suppositions only, when, by that very course, I have raised the number far beyond the reality, and have thus caused the comparison to be less in favour of London than in fact it is.

6. I have remarked that the number, in the relèves of Paris, reported drowned, is much larger annually than in the Bills of Mortality of London; and that, considering how much less the inhabitants of the former are occupied on the water, and exposed to this casualty, than those of the latter city, I have inferred the probability of the majority having met a voluntary death. (*Inq.* p. 89.) Dr. E. combats this inference, by stating that he has not verified the fact; but suspects the difference is owing, partly, to the greater solicitude of the English to bury their dead, and that, by this means, many drowned are privately interred, and are not entered under that head.

This opinion, however, as any Englishman would have informed him, is founded on ignorance of our laws and customs. A person privately burying a corpse that had been found, would be incurring a needless trouble and expence ; and would, besides, run the danger of being suspected as an accomplice in the death.

7. Further ; Dr. E. maintains that it would be a deception to decide that the medium of suicides in Paris is 300, because there were that number in 1817; for in that year an epidemic prevailed, when suicides are infinitely multiplied. The influence of some epidemics in producing self-destruction, is not to be disputed. But why does Dr. E. overlook the Paris official report in 1819, to which I have referred, (*Inq.* p. 90,) and even quoted? It is a memorable record: "In the four first months of 1819, the suicides (in Paris) amounted to 124; at the end of June, the number had arrived at 199!"

8. If he wish for further evidence, and from French authority too, of the great prevalence and increase of suicide in France, I advise him to read the petition presented, in May 1819, to the Chamber of Deputies, praying for the revival of the ancient law against this crime; and in which it is also stated, that four cases of suicide had occurred in one day in the city of Lyons! (*Inq.* p. 91.) Dr. E. particularly comments on one part of this paragraph, but finds it convenient to pass on without noticing the end of it, which contains this appalling statement.

9. In conclusion; Dr. Esquirol alleges that the acts of the British Parliament attest that, in 1815, there were, in London and its environs, 7000 lunatics; while in Paris there were not 3000. Now, as Dr. E. has often quoted it in his other writings, he is well aware that he derived this intelligence, not from an Act of Parliament, but from a printed Report of Evidence taken before a committee of the House of Commons. He knows, also, that it is there plainly put down, in question and answer, that Mr. Dunston, the superintendant of St. Luke's Hospital, being asked what number of lunatics *he imagined* there were in and about London, replied, "I really cannot exactly answer that question: I should *suppose*, 6 or 7000." (*Parl. Rep.* 1815, p. 127.)

Allowing Dr. E., as a foreigner, might have confounded a Report with an Act of Parliament, yet what is to be thought, when I have myself pointed out the vagueness and inaccuracy of Mr. Dunston's statement. (*Inq.* p. 96.) Moreover, I have particularly referred (*ibid.* p. 98,) to the parliamentary returns of the whole number of lunatics, in all the hospitals, public asylums, private licensed houses, and jails, in England and Wales, and have printed and appended them to the "Inquiry." Both in my text and appendix, therefore, Dr. E. must have seen that the total of lunatics in the kingdom (not London alone,) is officially reported to amount only to 4,041. Neither, if he had read what I have said, can he be ignorant that I have explained many deficiencies in these returns, (*ibid.* p. 98, 99,) and have stated my conviction that 6000 would be much nearer the truth than 4000.

However little Dr. E. may feel inclined to confess that there may be a period when, even "dans notre belle France," as Dr. Falret exults, a disposition to suicide is engendered, and exists in a greater degree than in Great Britain, others will acknowledge it. Had he been actuated by a sincere desire of establishing the truth, and had doubted my accuracy and the authorities I adduced, he might easily have obtained such information as to convince him that any attempt to confute my positions would be futile.

Dr. Falret I shall very briefly notice. He has adopted all Dr. Esquirol's errors, even in the title of my book; all his mistatements, intentional or unintentional; and, consequently, all his false inferences. He has likewise made others peculiar to himself. As an instance of his want of adherence to fact, and the *animus* with which he enters into the discussion, I will refer to his translation (*De l'Hypochondrie*, &c. p. 100, 101,) of a passage of mine, (*Inquiry*, p. 88, 89.) It will appear that, besides a material alteration in the text, similar to Dr. Esquirol's, he has most disingenuously substituted the year 1813 for 1817, the period of the comparison of the number of suicides in Paris and London; and then modestly accuses me of selecting a year most suitable to my own views, but the most unfavourable to Paris.

Had I really selected the year 1813, instead of 1817, for the period of comparison, Dr. Falret would have found more reason to thank than blame me; for, though the former was a year of severe and unexampled trial to the French metropolis, yet the proportion of suicides was actually less than in 1817, or in any subsequent year.

My only reason for fixing on 1817 was because M. Kamptz, of Berlin, had arranged his comparative table of suicides in Germany upon the casualties of that year: I therefore selected the corresponding period as the fairest for the calculation, and comparison of suicides in other European cities.

Having so fully replied to Dr. Esquirol, it would be a work of supererogation to comment further on the errors and sophistry of Dr. Falret.

*Gower-street, Bedford-square;*
*October* 1822.

